# Quantitative Analysis of Phase Wave of Gene Expression in the Mammalian Central Circadian Clock Network

**DOI:** 10.1371/journal.pone.0023568

**Published:** 2011-08-26

**Authors:** Hirokazu Fukuda, Isao Tokuda, Seiichi Hashimoto, Naoto Hayasaka

**Affiliations:** 1 Department of Applied Life Sciences, Graduate School of Life and Environmental Sciences, Osaka Prefecture University, Sakai, Japan; 2 PRESTO, Japan Science and Technology Agency (JST), Kawaguchi, Saitama, Japan; 3 Department of Micro System Technology, Ritsumeikan University, Shiga, Japan; 4 Molecular Medicine Research Labs, Drug Discovery Research, Astellas Pharma Inc., Tsukuba, Ibaraki, Japan; 5 Department of Anatomy and Neurobiology, Kinki University School of Medicine, Osaka-Sayama, Osaka, Japan; Vanderbilt University, United States of America

## Abstract

**Background:**

The suprachiasmatic nucleus (SCN), the master circadian clock, is a heterogeneous oscillator network, yet displays a robust synchronization dynamics. Recent single-cell bioluminescent imaging revealed temporal gradients in circadian clock gene expression in the SCN *ex vivo*. However, due to technical difficulty in biological approaches to elucidate the entire network structure of the SCN, characteristics of the gradient, which we refer to as phase wave, remain unknown.

**Methodology/Principal Findings:**

We implemented new approaches, i.e., quantitative analysis and model simulation to characterize the phase waves in *Per2::Luciferase* clock reporter gene expression of the rat SCN slice. Our quantitative study demonstrated not only a high degree of synchronization between the neurons and regular occurrence of the phase wave propagation, but also a significant amount of phase fluctuations contained in the wave. In addition, our simulations based on local coupling model suggest that the intercellular coupling strength estimated by the model simulations is significantly higher than the critical value for generating the phase waves. Model simulations also suggest that heterogeneity of the SCN neurons is one of the main factors causing the phase wave fluctuations. Furthermore, robustness of the SCN network against dynamical noise and variation of the natural frequencies inherent in these neurons was quantitatively assessed.

**Conclusions/Significance:**

To our knowledge, this is the first quantitative evaluation of the phase wave and further characterization of the SCN neuronal network features generating the wave i.e., intercellular synchrony, phase fluctuation, strong local coupling, heterogeneous periodicity and robustness. Our present study provides an approach, which will lead to a comprehensive understanding of mechanistic and/or biological significance of the phase wave in the central circadian oscillatory system.

## Introduction

Biological clocks, the generators of the circadian rhythm with a natural period of nearly 24 h, are ubiquitous in almost all living organisms. In mammals, the master circadian clock is located in the suprachiasmatic nucleus (SCN) of the brain [1], [2], [3], [4], [5], [6]. In the rat SCN, at least two subregions have been reported, i.e., the ventrolateral SCN (vlSCN, core) and the dorsomedial SCN (dmSCN, shell). The vlSCN, which perceives light inputs from the retina and projects upon shell, comprises primarily the vasoactive intestinal peptide (VIP)-producing neurons and surrounding astrocytes. In contrast, neurons producing arginine vasopressin (AVP) are predominant in the dmSCN, which receives non-visual inputs from cortical/subcortical regions [7], [8], [9] and projects to a broader set of effector area than vlSCN [9]. Coordinated but not uniform neuronal interactions were demonstrated by temporal gradients in circadian clock gene (*Per1* and *Per2*) expression in the dmSCN *in vivo* [10]. The temporal patterns of gene expression were consistent with those of neuropeptide release from SCN slices [11], suggesting topological and functional compartmentalization in the SCN. The recent technology of bioluminescence imaging has further revealed synchronization of the SCN neurons and the robust temporal gradients in circadian clock gene expression in cultured SCN slices, which persist for weeks [12], [13], [14]. This kind of coordinated and recurring gradients, which we refer to as “phase wave propagation” in this study, potentially reflect unique and critical characteristics of the central circadian clock. Little is however known about the mechanism underlying the phase wave propagation or its mechanistic or biological significance as compared to homogeneous coupling [15], [16], [17], mainly due to technical limitations for comprehensive understanding of the entire neural network.

According to previous studies, dissociated SCN neurons exhibit independent circadian periods and phases [12], [13]. This implies that such heterogeneous neuronal activities are synchronized to form a coherent circadian output in the SCN. Possible candidates for intercellular coupling that induces synchronization in the SCN are neuropeptide VIP, neurotransmitters [9], [18], and gap junctions [19], [20]. For instance, it has been suggested that VIP expressed in the vlSCN and its receptor VPAC2 play a crucial role both in sustaining the circadian rhythmicity and in synchronizing the SCN neurons [21], [22], [23], [24], [25], [26]. Neuroanatomical studies indicated that the SCN neurons, especially in the dmSCN, are tightly interconnected via somato-somatic apposition, resulting in ephaptic (non-synaptic) interaction [27]. This unique morphology and intercellular interaction raise the possibility that the phase wave in the SCN is generated and sustained by specific neural circuits including “local coupling” in the SCN. It should also be noted that the phase waves are robust and are observed in a consistent manner under various experimental conditions, suggesting that the intercellular coupling is sufficiently strong, although the coupling strength has not been quantitatively assessed.

The single-cell bioluminescence imaging technique provides a strong tool to reveal quantitative characteristics of the spatiotemporal dynamics of the whole measured area [28]. For instance, this technique has shown that circadian oscillators in a plant leaf produce a variety of nonlinear spatiotemporal patterns such as spiral waves [29]. Quantitative analysis of the imaging data may provide insight into network characteristics of the SCN such as interaction and coupling strength between the circadian oscillators.

Here, to characterize the network structure of the SCN, we investigated the spatiotemporal dynamics of the entire area of rat SCN slices using a highly-sensitive bioluminescence imaging technique. Based on the data analysis, fluctuations of the phase waves in the SCN slices were extracted and a simple mathematical model that simulates the experimental data was constructed.

## Results

### Bioluminescence of cultured SCN slices from *Per2::Luc* transgenic rats

**Figure 1:**
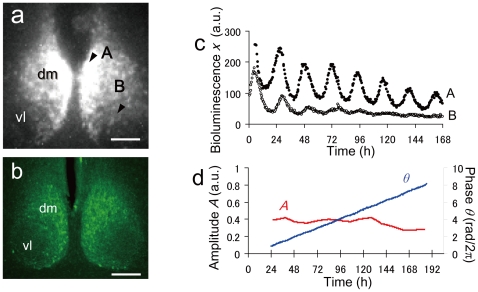
Oscillatory bioluminescence of a cultured rat SCN slice from a *Per2::Luc* transgenic rat. (a) A bioluminescence image of the cultured SCN slice. (b) Image of an immunostained SCN section. AVP (arginine vasopressin)-positive neurons predominantly distributed in the dmSCN are shown. The scale bar indicates 200 µm length. (c) Time series of bioluminescence *x(t)* at points (A) and (B) indicated in Fig. 1a. (d) Amplitude *A(t)* (red line) and phase *θ(t)* at point (A) computed, respectively, by Eq. [1,2]. dm: dorsomedial SCN; vl: ventrolateral SCN.

Fig. 1a shows a single-cell bioluminescence image of a cultured SCN slice from a *Per2::Luc* transgenic rat. Fig. 1b demonstrates AVP immunostaining of a rat SCN section for rough indication of the dmSCN and vlSCN. The intensity of the bioluminescence in the dmSCN (A, Fig. 1a) was higher than that in the vlSCN (B, Fig. 1a), while the oscillation amplitude in the dmSCN was also higher than that in the vlSCN (Fig. 1c). In both regions, the neurons showed damped oscillations *ex vivo*. Fig. 1d shows the amplitude *A* and phase *θ* of the oscillatory bioluminescence on the SCN slice (see *Materials and Methods*). *A* and *θ* extracted in the present study were almost equivalent to *A_H_* and *θ_H_* obtained by the Hilbert transform [30], suggesting that the phase of the experimental data was well defined and consistent with the two different transforms.

### Spatiotemporal variation of phase and amplitude in cultured SCN

**Figure 2:**
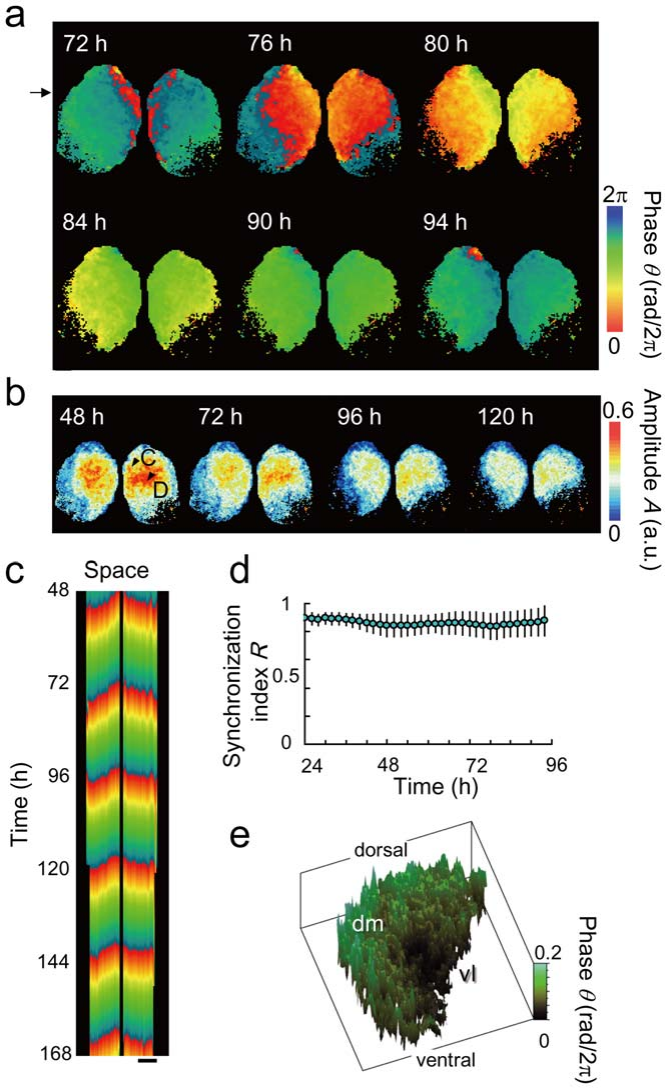
Spatiotemporal variation of the phase and amplitude of a cultured SCN slice. (a) Time evolution of the phase *θ*. (b) Time evolution of the amplitude *A*. The elapsed time from the beginning of the measurement is indicated in each image. (c) Space-time plot of phase *θ* on the horizontal line indicated by the arrows in Fig. 2a. The scale bars correspond to 200 µm length. (d) Synchronization index *R* computed by Eq. [5] for four different SCN slices, indicating strong synchronization among SCN neurons. Error bars indicate the standard deviation. (d) Three-dimensional representation of the phase pattern with spatial fluctuation on the right-side SCN slice at *t* = 99.5 h.

Fig. 2a–d shows the spatiotemporal variation of *θ* and *A* in the SCN slice. The phase waves were initiated from the innermost dmSCN and traveled regularly from the dmSCN to the vlSCN with a velocity of about 0.2 mm/h (Fig. 2a, see Video S1). Interestingly, the maximum oscillation amplitude was observed at the center of the dmSCN (Fig. 2b). The bioluminescence intensity at the innermost SCN (C) was higher than that at the central SCN (D), while the amplitude of detrended and normalized bioluminescence at (C) was smaller than that at (D). The phase wave propagations were symmetric between the right and left areas of the slice (Fig. 2c). To address the synchronization of the SCN quantitatively, the synchronization index *R*, which is known as the order parameter [31], was introduced as
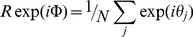
(5)where *θ_j_* represents phase of the *j*th pixel, *N* is the pixel number of the phase image, and Φ indicates the phase of the entire set of oscillators in the whole area. The synchronization index *R* takes a real value between 0 and 1, where a large value close to *R* = 1 indicates strong synchronization and a small value close to *R* = 0 indicates desynchronization. In the measured duration, the synchronization index *R* constantly indicated a high value of *R*∼0.85 (Fig. 2d), suggesting a strong synchronization among the SCN neurons.

### Spatial fluctuations of bioluminescence and phase on the surface of cultured SCN

Due to spatial fluctuations observed in the phase plane, the front of the phase wave had a discontinuous structure (Fig. 2a). The extent of this spatial fluctuation *Δ* can be measured by the standard deviation of the spatially detrended phase *θ* where *θ* is obtained by removing the locally averaged phase of the neighboring 7×7 pixels (equivalent to 3×3 cells) from *θ* [32], [33] (see Text S1). In our experiments, *Δ* was computed as 0.017+/−0.003 rad/2*π* for six regions of three different SCN slices, corresponding to a spatial fluctuation of about 25 min (1.7% of the circadian period). This fluctuation is relatively large compared to the maximum phase difference of about 0.2 rad/2*π* (about 5 h) observed between the starting point and the end point of the phase wave propagating in the SCN slice (Fig. 2a). Fig. 2e shows a three-dimensional representation of the phase pattern, which corresponds to that in the right-side of SCN in Fig. 2a. The fluctuation pattern was stable, indicating that the spatial fluctuation of the phase pattern is due to an inherent property of the SCN network.

### Mathematical Modeling

#### Estimation of the intercellular coupling strength

**Figure 3:**
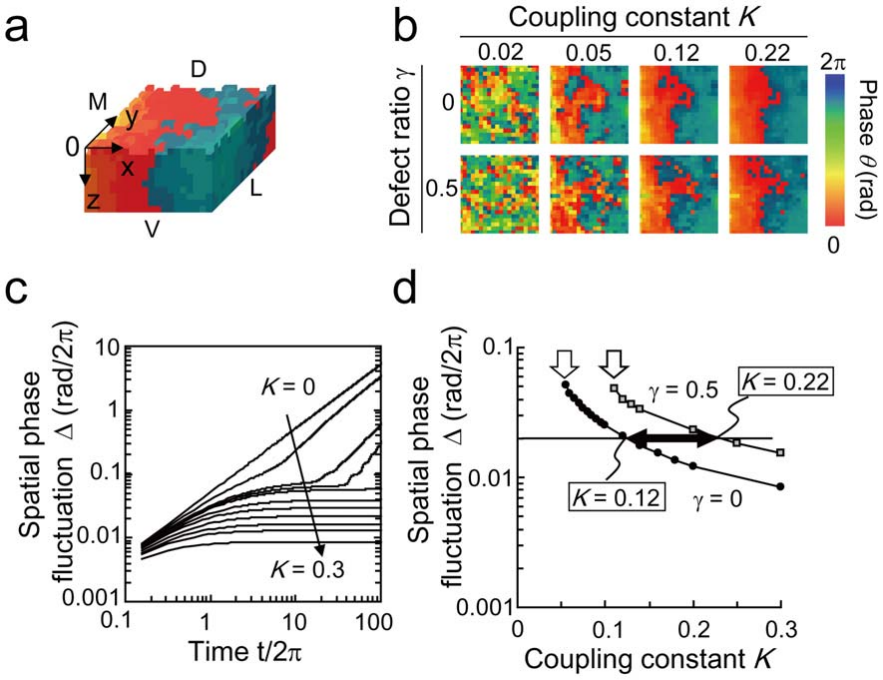
Estimation of the intercellular coupling strength by the model simulations. (a) Phase image of the SCN slice with a multi-layer structure. The pacemaker is located on the x = 0 plane, where oscillation frequency of the cells is 4% faster than that of the neurons in the other regions. The directions of ventral (V)/dorsal (D) and medial (M)/lateral (L) have been illustrated. The color map is the same as in Fig. 2a. (b) Phase patterns on the surface of SCN (z = 0). (c) Time evolution of *Δ* for various values of *K* (from top to bottom, *K* = 0, 0.02, 0.04, 0.045, 0.05, 0.07, 0.09, 0.12, 0.16, 0.2, 0.3). (d) *Δ* as a function of *K*. White arrows indicate the critical value of *K_c_*. Black arrow indicates the coupling strength region estimated for experimental SCN. The parameters were set as *N_L_* = 10 and *σ_ω_* = 0.05.

To gain more insight into our experimental observation of the phase wave propagation, numerical simulations were performed using a model of coupled phase oscillators:
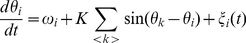
(6)where *θ_i_* represents the phase of the *i*th neuron, *ω_i_* represents natural frequency of the *i*th neuron, which is distributed normally with average <*ω*> and standard deviation *σ_ω_*, and *K* stands for the coupling constant. The summation over the nearest neighbors of the *i*th neuron is denoted by *<k>*. Our model has a multi-layer structure composed of *N_1_* width×*N_2_* length×*N_L_* layers (Fig. 3a). The surface (z = 0) of the present system contains many defects, which are regarded as extinct neurons in our simulations. The defects were randomly created with a probability of *γ*. Moreover, oscillation frequencies of the neurons on the x = 0 plane, which initiate the oscillation in the dmSCN, were set to be 4% faster than those of the vlSCN neurons as assumed in the previous modeling work [34], which takes into account various experimental conditions such as [35] (see Text S1). Thus, the natural frequencies of the pacemaker neurons were set as 1.04*ω_i_*. In addition, dynamical noise *ξ_i_(t)* was introduced by independent white Gaussian <*ξ_i_(t)*> = 0, <*ξ_i_(t) ξ_j_*(*s*)> = 2*Dδ_ij_*(*t-s*). Numerical simulations were performed using the fourth-order Runge-Kutta method with a time step of 0.02. The parameter values were set as *N_1_* = 20, *N_2_* = 20, <*ω*> = 1. For all simulations, a completely synchronized state was used as the initial condition (*θ_i_*(*t* = 0) = 0 rad).

Fig. 3b shows phase patterns on the surface of the SCN (z = 0). The phase of the defect on the surface was replaced by the phase of the inside oscillator under that defect. This manipulation corresponds to our experiments, which indicate bioluminescence of the inside neurons as background. As the coupling strength reaches to the critical value *K_c_*, which provides minimal coupling strength to maintain synchronization, all neurons on the surface were synchronized and phase waves were formed. The phase wave traveled from left (x = 0), where the oscillation is initiated, to right. Fig. 3c shows the time evolution of spatial phase fluctuation *Δ* plotted for various values of *K*. In our simulation, *Δ* was defined as the standard deviation of the spatially detrended phase *θ* where *θ* was obtained by removing the spatial average of the neighboring 3×3 oscillators from *θ*. For *K*<*K_c_*, *Δ* increases in time. On the other hand, for *K*≥*K_c_*, *Δ* remains constant after an initial transient. Fig. 3d shows the relationship between *Δ* and *K* in the cases of *γ* = 0 and *γ* = 0.5. We see that *K_c_* depends on the defect ratio *γ*, e.g., *K_c_* = 0.05 for *γ* = 0 and *K_c_* = 0.11 for *γ* = 0.5. The spatial fluctuation of *Δ*∼0.02 rad/2*π* obtained from our experiment is indicated by the dotted line in Fig. 3d. This implies that the coupling strength of the experimental system lies between *K* = 0.12 and 0.22, which is approximately 2 to 4 times larger than the minimum coupling strength (*K_c_* = 0.05) required for the formation of the phase wave.

#### Spatial fluctuation of phase wave

**Figure 4:**
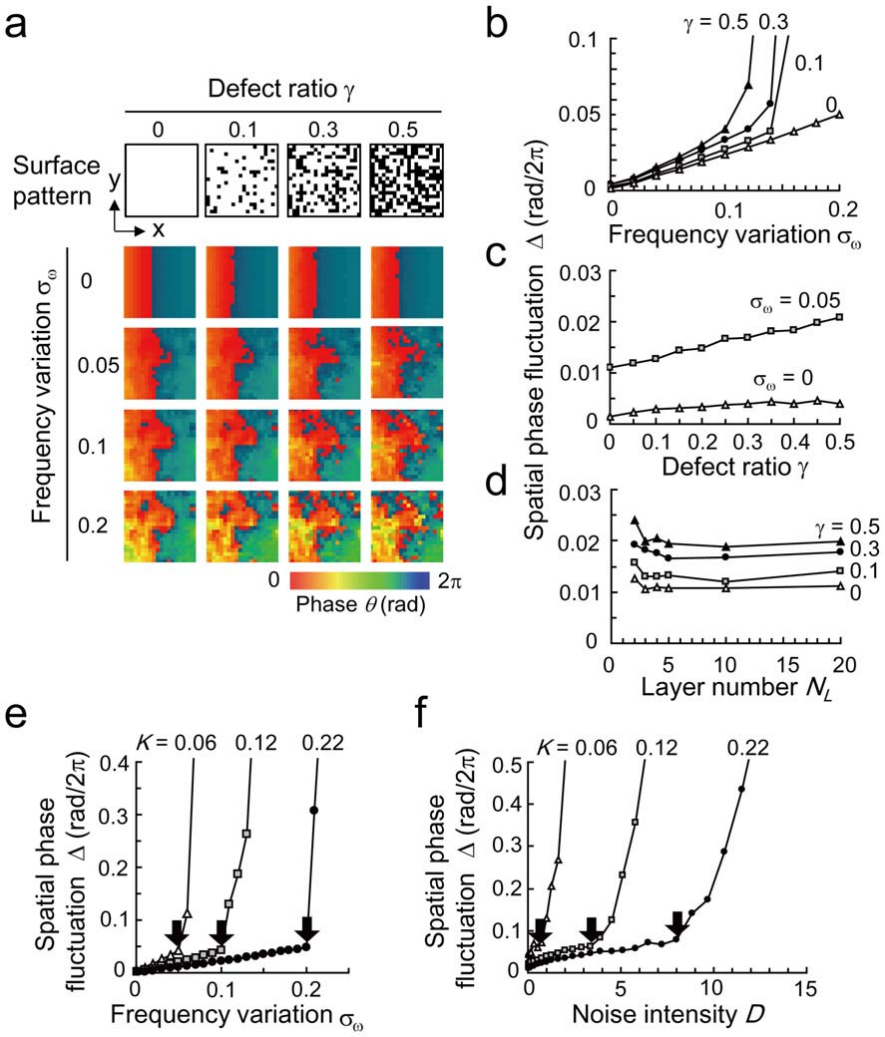
Spatial fluctuation and robustness of the SCN networks. (a) Dependence of the spatial phase fluctuation *Δ* on the standard deviation of the natural frequencies *σ_ω_* and the defect ratio *γ*. Coupling strength and number of the layers were set to *K* = 0.22, *N_L_* = 10. The color map is the same as in Fig. 2a. (b) Dependence of *Δ* on *σ_ω_* for *γ* = 0, 0.1, 0.3, 0.5. *K* = 0.22, *N_L_* = 10. (c) Dependence of *Δ* on *γ* for *σ_ω_* = 0, 0.05. *K* = 0.22, *N_L_* = 10. (d) Dependence of *Δ* on *N_L_* for *γ* = 0, 0.1, 0.3, 0.5. *σ_ω_* = 0.05, *γ* = 0.5, *K* = 0.22. (e) Dependence of *Δ* on *σ_ω_* for *K* = 0.06, 0.12, 0.22. *N_L_* = 10, *γ* = 0, *D* = 0. (f) Dependence of *Δ* on the dynamical noise intensity *D* for *K* = 0.06, 0.12, 0.22. *N_L_* = 10, *γ* = 0, *σ_ω_* = 0.05. Robust synchronization was observed up to the critical points indicated by the black arrows in (e, f).

Fig. 4a–d shows the dependence of the spatial phase fluctuation *Δ* on frequency variation *σ_ω_*, defect ratio *γ*, and layer number *N_L_*. Among them, the frequency variation has the strongest influence on *Δ*. Although the influence of *γ* on *Δ* is smaller than that of *σ_ω_* there exists a multiplicative effect between *σ_ω_* and *γ*. Namely, the influence of *γ* on *Δ* increases with *σ_ω_* (Fig. 4a, c). In addition, *Δ* is almost constant for *N_L_*>3, whereas *Δ* increases in the case of *N_L_* = 2, which is composed only of surface and background layers (Fig. 4d). This suggests that the spatial phase fluctuation remains constant except for such extremely thin slices. This could explain why similar phase waves can be observed in SCN slices with various thicknesses. Our model also suggests that the thickness of the cultured SCN slice has a strong influence on the degree of synchronization. In fact, circadian rhythms of thin SCN slices (e.g., ≤100 µm thickness) have a tendency to show a weak degree of synchronization and they are dampened earlier than the thick ones (Fig. S2).

#### Robustness of the SCN networks

Finally, we investigated the robustness of the SCN dynamics against heterogeneity and dynamical noise of the SCN neurons. Robustness is defined as the network configuration required to maintain synchronized neuronal activities. Fig. 4e shows the dependence of the spatial fluctuation *Δ* on the standard deviation of the natural frequencies *σ_ω_*. Synchronized dynamics indicated with finite *Δ* has been observed up to *σ_ω_* = 0.05, 0.1, and 0.2 for *K* = 0.06, 0.12, and 0.22, respectively, which indicates that the robustness against *σ_ω_* increases proportionally to *K*. Fig. 4f, on the other hand, shows robustness of the network synchrony against dynamical noise *D*. For *K* = 0.06, 0.12, and 0.22, synchronized dynamics has been observed up to *D* = 0.7, 3.4, and 8, respectively, indicating that the robustness against noise *D* increases more rapidly than that against *σ_ω_*. Therefore, our simulations suggest that the synchronized dynamics is more robust against noise than against heterogeneity of the neurons.

## Discussion

In the present study, we analyzed the spatiotemporal dynamics of the entire area of SCN slices for quantitative characterization of the phase waves in the SCN. First, our quantitative analysis indicated a high synchronization index of about 0.85 (∼85% synchrony), suggesting a strong synchronization among the SCN neurons. We also demonstrated that the phase waves, which regularly traveled from dmSCN to vlSCN, included a significant amount of fluctuations. The largest amplitude was observed not in the innermost dmSCN, where *Per2* oscillation is initiated, but in the central dmSCN. This discrepancy might be due to a structural property of the central dmSCN (e.g., a difference in cell density or cell-cell interaction), in which small and densely distributed neurons are connected via non-synaptic interaction [27].

To elucidate the experimental observations, we constructed a network model of coupled phase oscillators based on a local coupling and performed numerical simulations. The purpose of our modeling is to characterize the phase wave propagation, spatial phase fluctuations and phase synchronization in the central region of the dorsal SCN as shown in Figs. 3b and 4a. The distribution of amplitude in the central region was flat as shown in Fig. 2b, although there is a large gradient of amplitude from the center to peripheral regions. Therefore, the neuronal network in the central SCN region can be described by phase equations. We found that the intercellular coupling strength for cultured SCN slices was estimated to be significantly higher than the critical coupling strength for generating the phase waves. Our study also showed that strong coupling realized robust synchronization against dynamical noise and network heterogeneity. In addition, our simulations suggested that phase wave fluctuations in the SCN are primarily caused by frequency variation, which reflect variability of period length among SCN neurons [34], [35]. Finally, analysis of the robustness of the SCN dynamics suggested that the synchronized dynamics is more robust against noise than against heterogeneity of the neurons.

One possible explanation for the phase wave fluctuation may be neuronal damage on the SCN surface during *ex vivo* slice preparation/culture. However, this may not be the case, because robust circadian rhythmicity has been observed in SCN slices for several months under different experimental conditions. Instead, the phase fluctuation could be due to the heterogeneous structure inherent in the SCN network, since discontinuous phase waves cannot be observed in a network of homogeneous oscillators such as the Belousov-Zhabotinsky reaction [36]. Individual SCN neurons are known to show a large variation in their natural frequency (5% distribution) [35], [37], [38]. Moreover, other studies report different types of SCN neurons with distinct characteristics in morphology and electrophysiology [27], [39]. Therefore, our present data, along with previous observations, raise a possibility that the spatial phase fluctuation is generated by such heterogeneity of the SCN neurons.

From a biological viewpoint, our present study raises the question of whether formation of the phase waves plays a significant role in the SCN. One possible interpretation is that the dispersal of phase is due to “phase lag”, which is the outcome of sequential interaction of thousands of locally coupled cells within the dmSCN. Based on our data indicating the velocity of the phase plane (∼0.2 mm/h) in the dmSCN, where small neurons (∼10 µm) are tightly apposed, a rough estimate is that each cell-to-cell transmission of phase information takes anywhere from several to 10 min. Although this lag results in about a 5-h phase difference between the innermost and outermost dmSCN subpopulations, there might still be a mechanism to form a coherent circadian output as long as the synchronization index is high (Fig. 2d). Alternatively, there is the possibility that the phase wave reflects an ordered progression of gene activation that could regulate distinct subgroups of neurons to transmit differentially-phased output signals. This may partly explain previous observations in which different brain regions exhibit different phases in circadian clock gene expression [40]. To test this hypothesis, detailed analyses of efferent signals from the dmSCN will be required. Thirdly, phase wave formation may have been developed as a necessary or advantageous property of the SCN circuitry. As already mentioned, the phase wave produces a significant amount of phase lag between the innermost and outermost dmSCN. Such phase lag may provide robustness against phase shift induced, e.g., by jet-lag in the sense that it can adapt to different circadian phases through desynchronization and following resynchronization.

In summary, our quantitative analysis and mathematical model simulation of the phase wave propagation provided new insights into the SCN neural network structure: i) high synchronization index, ii) significant phase fluctuations caused by heterogeneity of the SCN neurons, iii) significantly high intercellular coupling strength, and iv) robustness against noise and heterogeneity of neurons. These data based on local coupling model will provide advanced understanding of phase wave propagation *in vitro* and *in vivo*, although biological approaches elucidating precise cell-cell interaction and morphology of the entire neural network in the whole SCN is still difficult. Novel biological tools would be required to further analyze the detailed network structure and involving factor(s) allowing this well-coordinated and synchronized oscillatory system, e.g., genetically engineered mice that demonstrate abnormal/ablated phase waves or specific chemicals that could alter the pattern of normal phase wave propagation by changing intercellular communication. To this end, mice with genetic manipulation of the *Rgs16* gene (regulator of G protein signaling), which is robustly expressed in the SCN in a circadian manner, could potentially be a powerful tool to address unsolved questions regarding the phase waves in the SCN [41], [42]. A recent report demonstrated that *Rgs16* knockout mice exhibited abnormal phase wave of the *Per2::luc* in the SCN, as well as altered locomotor activity rhythms [42]. Studies on these animals could elucidate biological significance of the phase wave by studying outputs of altered or disrupted phase wave.

In terms of mathematical approach, more detailed modeling of the coupled circadian oscillators will be required in the future study. Since the SCN neurons interact not only with neighboring neurons but also with distant neurons through diffusible factors [26], [43], non-local and global coupling should be considered. It has also been speculated that there exists a monotonic gradient in the spatial distribution of the natural frequencies of the oscillators in the dmSCN. Such gradient may strongly contribute to the formation of the phase wave even under the global coupling. The effect of the gradient distribution on the phase wave should be considered. Finally, we should also take into account the fact that the network structure of the SCN differs by species, developmental stage, and age.

## Materials and Methods

### Transgenic rats

Transgenic rats (Wistar, Charles River) carrying a *Per2::Luciferase* reporter gene were generated as described previously [44]. Briefly, 3510 bp of the mouse *Per2* promoter region was fused to a destabilized luciferase (*dLuc*) reporter gene (firefly luciferase gene fused with a modified PEST sequence [45]). The *Per2::dLuc* transgenic rats were generated in accordance with the method described in patent publication number WO/2002/081682 (PhoenixBio). All experiments were conducted following the guidelines for the care and use of laboratory animals of Kinki University School of Medicine (Permission #KDMS-16-002).

### Explant cultures and bioluminescence measurement

All rats were kept under 12 h light∶ 12 h dark (12∶ 12 LD) condition. Coronal brain slices including the SCN (300 µm thickness) were prepared from 2-week-old rats (n = 5). Brain slices including paired SCN were explanted from the coronal brain sections and placed on a culture membrane (Millicell-CM, PICM030-50; Millipore) in a covered and sealed petri dish, and cultured with DMEM-F12 (Sigma) supplemented with B27 (Gibco-Invitrogen) and D-luciferin (Nacalai Tesque). Bioluminescence rhythm of each SCN slice was monitored by a luminometer (Kronos, ATTO) for two days, followed by single-cell bioluminescence imaging with a luminescence microscope optimized for live cell imaging (LV200, Olympus) as described previously [28].

### Immunohistochemistry

Brains from two-week-old rats were fixed with 4% paraformaldehyde for overnight at 4°C. Frozen brain sections (coronal, 30 µm thickness) were incubated for 4 days at 4°C with anti-AVP antibodies (Chemicon) diluted 1∶10,000 in PBS. The sections were rinsed and incubated with fluorophore-labeled anti-rabbit or anti-mouse IgG antibody (Alexa Fluor 488; Invitrogen) diluted 1∶1000 in PBS. The slices were rinsed and embedded in gel/mount (Biomeda), and imaged using a laser scanning confocal microscope (Zeiss).

### Amplitude and phase analysis of the oscillatory bioluminescence

To quantify the spatiotemporal dynamics of the circadian oscillations in the cultured SCN slices, we introduced amplitude *A(t)* and phase *θ(t)* of the bioluminescence oscillations *x(t)* in each pixel as
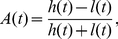
(1)

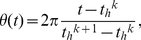
(2)where *h(t)* and *l(t)* indicate peak and bottom envelopes, respectively, calculated by linear interpolation of the peaks (highest emission) and the bottoms (lowest emission) of each oscillation as
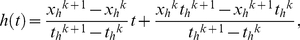
(3)

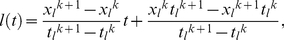
(4)where *x_h_^k^* and *x_l_^k^* represent bioluminescence at the *k*th peak and *k*th bottom of the time series in each pixel, *t_h_^k^* and *t_l_^k^* stand for occurrence time of the *k*th peak and *k*th bottom, and *t_h_^k^*≤*t*<*t_h_^k+1^* and *t_l_^k^*≤*t*<*t_l_^k+1^*. To remove the noise included in the bioluminescence data, moving average filter with 6 h window size was applied. Then peaks and troughs were identified as local maxima and local minima of the smoothed data, respectively.

## Supporting Information

Figure S1 **Spatial fluctuations of bioluminescence and phase on the surface of cultured SCN slice.** (a) Intensity of bioluminescence *x* during a circadian cycle (48 h to 72 h). The white rectangle corresponds to the region enlarged in Figs. S1b and S1c. (b), (c) Binary patterns of the spatially detrended bioluminescence *δx* and phase *δθ* at *t* = 58, 82, 106 h. White color indicates *δx*>0 or *δθ*>0, whereas black color indicates *δx*<0 or *δθ*<0.
(TIF)

Figure S2 ***Per2::Luc*** **bioluminescence rhythms in thin SCN slices.** Representative *Per2::Luc* rhythms in the SCN slices at the thickness of 150 µm (a) and 100 µm (b), respectively. Note that the amplitude of the *Per2::Luc* fluctuation is significantly lower and the rhythmicity does not persist long in (b) as compared to (a). Bioluminescence intensities are comparable between the two slices, suggesting that the discrepancy is not caused either by slice preparation or culture condition.
(TIF)

Figure S3 **Dependence of spatial phase-fluctuation on the natural frequency of pacemaker.** (a) Phase patterns on the surface of SCN (z = 0) with *γ* = 0. The pacemaker is located on the x = 0 plane, where oscillation frequency of the cells is 4% (upper panels) or 2.7% (lower panels) faster than that of the neurons in the other regions. The color map is the same as in Fig. 3b. (b) *Δ* as a function of *K*, when the oscillation frequency of pacemaker is 4% (closed circles) and 2.7% (open squares) faster than that of the neurons in the other regions. The parameters were set as *N_L_* = 10 and *σ_ω_* = 0.05.
(TIF)

Video S1 This animated movie shows the spatiotemporal variation of bioluminescence *x* and the phase *θ*, amplitude *A* on the right side of the cultured SCN slice as shown in Fig. S1a for *t* = 30 h to136 h. The phase wave with the discontinuous wave front propagated from the dmSCN to the vlSCN. The innermost dmSCN region, in which the *Per2* oscillation is initiated, does not correspond to the area with the maximum oscillation amplitude. Instead, the area with the maximum amplitude was located at the center of the dmSCN.
(AVI)

Text S1 (DOC)
