# Supplementary figures and images for: Quantitative Analysis of Phase Wave of Gene Expression in the Mammalian Central Circadian Clock Network
Source: PLoS One. 2011 Aug 26;6(8):e23568. doi: 10.1371/journal.pone.0023568 (PMC3162606; doi:10.1371/journal.pone.0023568)

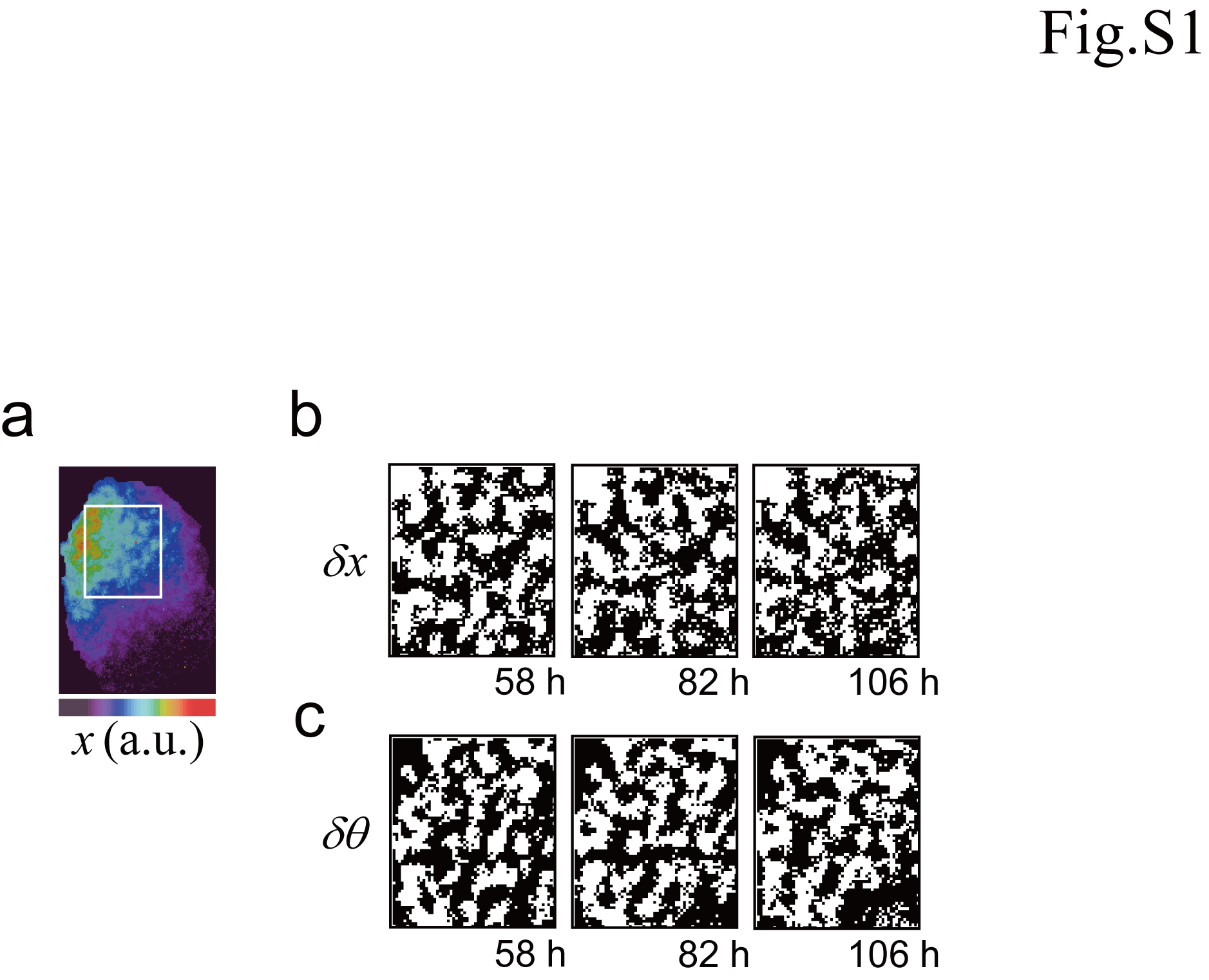

Supplement: Figure S1 — Spatial fluctuations of bioluminescence and phase on the surface of cultured SCN slice. (a) Intensity of bioluminescence x during a circadian cycle (48 h to 72 h). The white rectangle corresponds to the region enlarged in Figs. S1b and S1c. (b), (c) Binary patterns of the spatially detrended bioluminescence δx and phase δθ at t = 58, 82, 106 h. White color indicates δx>0 or δθ>0, whereas black color indicates δx<0 or δθ<0. (TIF) [file pone.0023568.s001.tif]

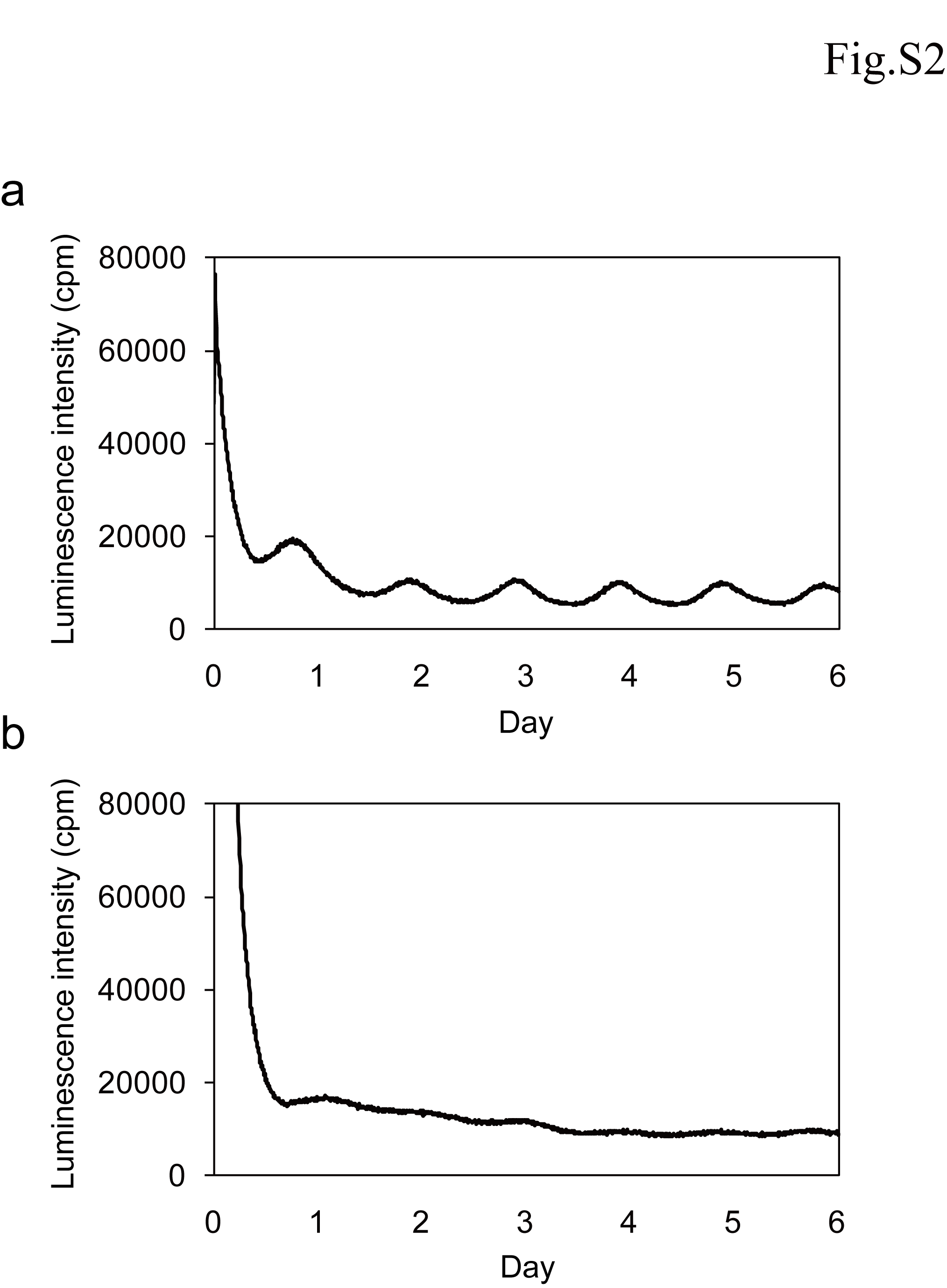

Supplement: Figure S2 — Per2::Luc bioluminescence rhythms in thin SCN slices. Representative Per2::Luc rhythms in the SCN slices at the thickness of 150 µm (a) and 100 µm (b), respectively. Note that the amplitude of the Per2::Luc fluctuation is significantly lower and the rhythmicity does not persist long in (b) as compared to (a). Bioluminescence intensities are comparable between the two slices, suggesting that the discrepancy is not caused either by slice preparation or culture condition. (TIF) [file pone.0023568.s002.tif]

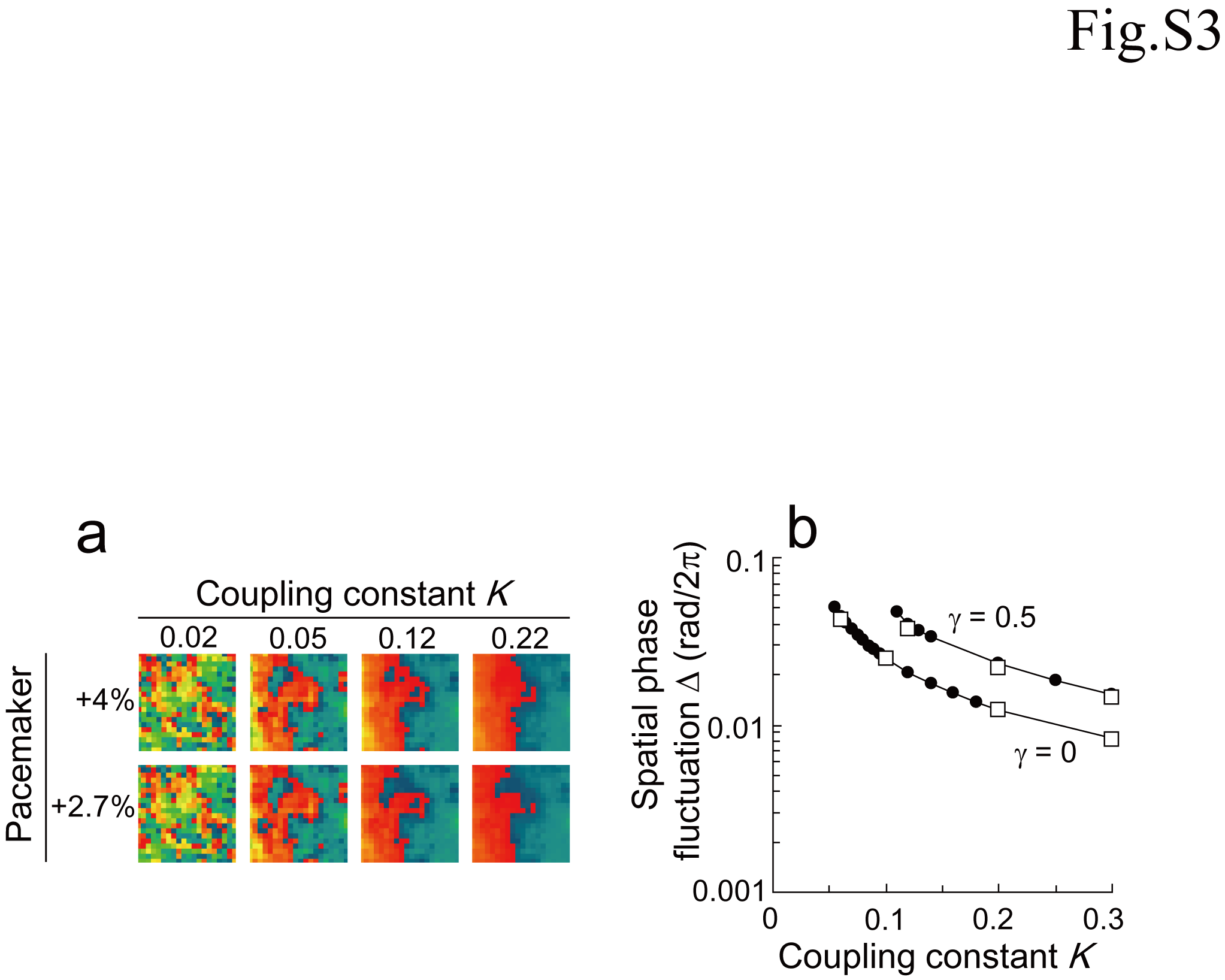

Supplement: Figure S3 — Dependence of spatial phase-fluctuation on the natural frequency of pacemaker. (a) Phase patterns on the surface of SCN (z = 0) with γ = 0. The pacemaker is located on the x = 0 plane, where oscillation frequency of the cells is 4% (upper panels) or 2.7% (lower panels) faster than that of the neurons in the other regions. The color map is the same as in Fig. 3b. (b) Δ as a function of K, when the oscillation frequency of pacemaker is 4% (closed circles) and 2.7% (open squares) faster than that of the neurons in the other regions. The parameters were set as NL = 10 and σω = 0.05. (TIF) [file pone.0023568.s003.tif]
