# Supplementary material for: Quantitative Analysis of Phase Wave of Gene Expression in the Mammalian Central Circadian Clock Network
Source: PLoS One. 2011 Aug 26;6(8):e23568. doi: 10.1371/journal.pone.0023568 (PMC3162606; doi:10.1371/journal.pone.0023568)
Supplement: Text S1 — (DOC) [file pone.0023568.s005.doc]

**Supporting Information**

*Analysis of the spatial phase-fluctuation*

To investigate the dependence of the spatial phase-fluctuation on the arrangement of neurons on the surface, we compared the spatial fluctuations between bioluminescence intensity and phase. Figure S1a shows bioluminescence intensity *x* during a circadian cycle, whereas Figures S1b and S1c show, respectively, binary patterns of the spatially detrended bioluminescence *δx* and phase *δθ*, which were obtained by removing the spatial average of neighboring 7 x 7-pixels from *x* and *θ* (The size of 7 x 7-pixels was chosen to take an average of the nearest neighbor cells because of the following reason. To take into account the nearest neighbor cells on a plane lattice, 3 x 3-cells should be grouped. The size of a single SCN cell is about 10 μm in length, whereas the size of one pixel is 4 μm in length. Therefore, 3x3 cells correspond roughly to 7x7 pixels). The area of *δx* > 0 was regarded as clusters of neurons on the surface, whereas the area of *δx* < 0 was considered as neurons inside of the slice. We consider that the bioluminescence intensity is strong for neurons located on the surface, which results in *δx>0*. On the other hand, neurons under the surface may show relatively weak bioluminescence, which may correspond to *δx<0.* We also note that our microscope focuses mainly on the surface neurons and neurons within a shallow depth. Hence, the bioluminescence image shows neuronal activities of up to few layers. Since bioluminescence from deeper layers has only a weak output, it can be considered as a spatially uniform background noise. In this sense, the pattern of *δx* indicates the arrangement of neurons on the surface. Correlation coefficient between the binary patterns of *δx* and *δθ* was small (correlation coefficient was estimated to be 0.06 +/- 0.07; *n = 6*). This implies that the spatial phase-fluctuation is not directly related to the arrangement of the neurons on the surface. In addition, the patterns of both *δx* and *δθ* were stable, indicating that such spatial fluctuations are due to an inherent property of the SCN network. We have investigated the stability by computing the correlation of the binary pattern of *δx* (and *δθ*) between two time points. The correlation values were 0.53 (images at *t* = 58 h and 82 h in Fig. S1 (b)), 0.57 (images at *t* = 82 h and 106 h in Fig. S1 (b)), 0.46 (images at *t* = 58 h and 82 h in Fig. S1(c)), 0.47 (images at *t* = 82 h and 106 h in Fig. S1 (c)). These relatively high correlations indicate that the timescale of the pattern transition is small compared to the circadian period.

*Computing synchronization index among cells*

The synchronization index was calculated using the phase data obtained from each pixel. Although the correlation between the neighboring pixels might slightly increase due to the overlapping effect of the bioluminescence from the nearby cells, it is expected that such influence becomes sufficiently small, because the phase of neighboring cells might be either slightly advanced or delayed. Overall, such an effect might be cancelled with each other especially by averaging of many neighboring cells. Moreover, the synchronization index computed from individual pixels should become asymptotically close to the one computed from individual cells, provided that the cell size is homogeneous among the SCN slices and the number of the cells used for the computation is large enough (~ 100 cells).

*Dependence of spatial phase-fluctuation on the natural frequency of pacemaker*

In the present study, oscillation frequency of the dmSCN was supposed to be 4% higher than that of the vlSCN according to Bernard *et al.* [34]. On the other hand, Noguchi *et al.* [35] reported the frequency difference of 2.7 % between dmSCN and vlSCN. Our model adopted the condition of Bernard *et al.*, since their assumption seems applicable to various experimental conditions. Since our experimental condition is not exactly the same as that of Noguchi *et al.*, we consider it is not necessary to strictly use the value of 2.7 %. For instance, Noguchi *et al.* used "knife-cut" dorsal SCN slices for measuring the average period, each of which may include different part of vlSCN as well as dmSCN. So the condition they determined the average period was different from ours. It should also be noted that the present result is not so sensitive to the value of the frequency difference. In fact, qualitatively the same results were obtained in the case of using the frequency difference of 2.7 % as shown in Fig. S3. In general, the pacemaker in locally coupled oscillator systems determines the traveling direction of the phase wave but has only a weak influence on either the traveling velocity of the phase wave or the spatial fluctuations of the phase in non-pacemaker region.
